# It’s Not All about Echocardiography. Open the Lung Window for the Cardiac Emergencies

**DOI:** 10.3390/medicina57010069

**Published:** 2021-01-14

**Authors:** Eftihia Polyzogopoulou, Antonios Boultadakis, Ignatios Ikonomidis, John Parissis

**Affiliations:** 1 Emergency Medicine Clinic, Attikon University Hospital, National and Kapodistrian University of Athens, 12462 Athens, Greece; boult_doc@yahoo.gr (A.B.); jparissis@yahoo.com (J.P.); 2 2nd Department of Cardiology, Attikon University Hospital, National and Kapodistrian University of Athens, 12462 Athens, Greece; ignoik@gmail.com

**Keywords:** lung, ultrasonography, cardiac, emergencies, differential, diagnosis

## Abstract

In the acute cardiac care setting, undifferentiated clinical presentations such as dyspnea, chest pain, shock, and cardiac arrest are common diagnostic challenges for the clinician. Lung ultrasonography is a well-established diagnostic tool which can be integrated in simplified decision making algorithms during the initial approach of the patient, in order to differentiate accurately cardiac from non-cardiac causes and improve the management of time-sensitive cardiovascular emergencies.

## 1. Introduction

In the emergency setting, patients usually present with symptoms, and rarely with established or direct recognized diagnoses. The life-threatening nature of the cardiac emergencies increases the diagnostic challenge as the patients are quite often unstable or in a peri-arrest condition and require rapid and accurate interventions, usually while resuscitation is ongoing.

Furthermore, it is well known that in common cardiac emergencies such as acute heart failure, acute coronary syndrome, and shock, physical examination and the routine work-up are often inconclusive, inaccurate, and misleading [1,2,3,4,5].

Although it has been practiced for more than 20 years starting from the intensivists [6], in the last decade, lung ultrasound has become a rapidly expanding sonographic application for specialties such as emergency physicians and pneumonologists [7,8]. Recently, it has become an emerging imaging modality for the cardiologists as well. Management of patients in the acute cardiac care setting is often challenging and obviously time-sensitive. The vast majority of those patients have comorbidities such as COPD, which present with a similar clinical picture, making the differential diagnostic puzzle even more complicated [9]. It is well known that a holistic assessment of acute cardiovascular pathology requires a multi-organ approach [10].

Nowadays, lung scanning is an essential component in the sonographic evaluation of the critically ill patient. Unlike the comprehensive echocardiographic study, lung scanning is a rapidly learned and immediately adopted modality which helps the cardiologist to narrow differential diagnosis and to improve his diagnostic accuracy in uncertain and complicated cases. Studies have shown that it is worthwhile implementing lung scanning in the management of patients with not clearly defined clinical presentations, as it has a clear impact on clinical decision making and patient’s outcome [11,12]. Even though its use is recommended by ESC (European Society of Cardiology) and the European Association of Cardiovascular Imaging (EACVI), a wide spread culture of its clinical utility hasn’t developed yet in the cardiologist’s world, and misconceptions regarding its use still exist [1,13].

This review outlines the clinical implementation of lung sonography during the initial management of various and the most common cardiac pathology-related symptoms. It focuses on the challenge to make a rapid and accurate differential diagnosis between cardiac and non-cardiac causes in the emergency setting in order to avoid dangerous treatment delays or wrong diagnostic and treatment pathways. As it shown in the next common clinical scenarios, whilst echocardiography remains the most commonly used initial imaging modality for the identification of cardiac pathologies, lung sonography becomes extremely useful in rule in or rule out of pulmonary diseases, which have a similar clinical picture with cardiac emergencies.

The scope of this review is to highlight the need of incorporation of lung sonography in the cardiologist’s everyday clinical practice, especially to those who manage critically ill and undifferentiated patients with cardiovascular emergencies.

## 2. Clinical Utility in Common Cardiac-Related Emergencies

### 2.1. Undifferentiated Dyspnea

Undifferentiated dyspnea is one of the most common and challenging clinical presentations due to its wide differential diagnosis. Life-threatening diagnoses of cardiac and non-cardiac origin, such as acute heart failure (AHF), pneumothorax, and pulmonary embolism, should be ruled in or ruled out as soon as possible. The main problem is that usually in the dyspneic critically ill patient, targeted therapy should be started based on assumptions even before any definite diagnostic clue. Electrocardiography, echocardiography, and natriuretic peptides can support the diagnosis of the cardiologic causes such as acute heart failure or acute coronary syndrome, but there is a diagnostic gap regarding the non-cardiologic causes of dyspnea. Integration of focused lung scanning by identifying the morphology and distribution of the different sonographic lung signs can rule in or rule out diagnoses, while initial management is ongoing.

**Figure 1:**
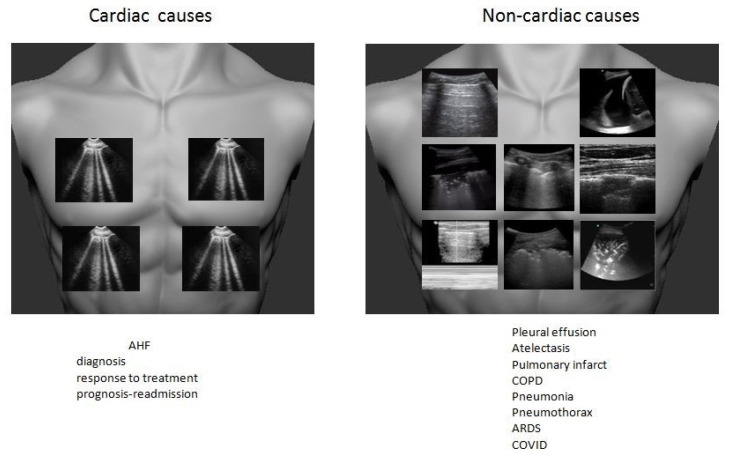
Lung algorithmic approach in undifferentiated dyspnea. AHF: acute heart failure; COPD: chronic obstructive pulmonary disease; ARDS: acute respiratory distress syndrome.

A simplified, algorithmic approach is illustrated in Figure 1.

The most widely implemented lung sonographic decision making tree is BLUE protocol, with a diagnostic accuracy of 90.4% [14]. According to Linchtestein, BLUE protocol includes scanning of upper and lower lung fields bilaterally. While checking for pulmonary congestion as the cause of dyspnea, the presence of horizontal artifacts, called A-lines, represent a dry-non congested lung pattern which is consistent with a non-cardiologic cause, like chronic obstructive pulmonary disease (COPD) or asthma. The presence of vertical, comet tail artifacts, depict a wet-congested lung pattern which is supportive of acute cardiogenic or non-cardiogenic pulmonary edema or an interstitial disease.

Acute heart failure is one of the most common cardiac emergencies and one of the main indications of lung scanning. Studies have shown that its diagnostic accuracy is superior to chest X-ray [15]. The appearance of multiple, diffuse distributed B-lines in the lungs bilaterally are an extremely useful index for the presence and the degree of congestion [16]. The diagnostic accuracy of lung sonography is excellent, with a sensitivity of 94% (95% CI 81–98%) and specificity of 92% (95% CI 84–96%) [17,18], whilst physical examination has a sensitivity of only 62% (95% CI 61–64%) and a specificity of 68% (95% CI 67–69%). Chest radiography has a sensitivity 57% (95% CI 55–59%) and a specificity of 89% (95% CI 88–90%) for a diagnosis of acute heart failure [19], and is normal in up to 20% of patients with acute heart failure [1]. Due to the great correlation of natriuretic peptides with lung sonographic findings, both can be used in conjunction in order to improve diagnostic accuracy [3,20]. In contrast, detection of multiple, heterogeneous bilateral B-lines which arise from an irregular and distorted pleural line make the diagnosis of acute heart failure unlikely. This sonographic pattern is more consistent with interstitial diseases such acute lung injury/acute respiratory distress syndrome (ARDS) and pulmonary fibrosis. Additionally, the presence of bilateral pleural effusions on lung scanning is a surrogate marker of acute heart failure [12]. For example, in a patient with respiratory distress, a focused triple scan of the lung, heart, and inferior vena cava with findings of bilateral, diffuse B-lines, bilateral pleural effusions, reduced left ventricular ejection fraction (LVEF) and a plethoric, non-collapsible inferior vena cava is highly indicative of acute heart failure. In contrast, in a patient with low pretest probability for acute heart failure, a normal lung sonography and normal LVEF almost excludes this diagnosis.

Lung ultrasound is not only a great diagnostic tool in acute heart failure, but it can be used as an alternative marker of pulmonary decongestion. It has the power to monitor dynamic changes in B-lines resolution in response to treatment [21,22,23] and predict readmission according to pre-discharge persistence of B lines [24,25]. Thus, the synergistic use of echocardiography and lung ultrasonography can be of great value for the early identification of acute heart failure especially in patients with respiratory comorbidities [25,26].

Additionally, in the acutely dyspneic patient, it is a complementary tool for the rapid identification of non-cardiologic causes. Hence, unilateral pleural effusion, consolidation pattern like an atelectasis or sonographic hepatization of the lung, and dynamic air bronchograms, which are indicative of pneumonia, absence of lung sliding and the presence of lung point, which represents pneumothorax, wedge-shaped consolidation like an infract, all depict acute lung pathology [27].

In the COVID-19 era, due to the undifferentiated clinical presentation and the multi-organ effects of the disease, cardiologists are frequently involved in the care of COVID-19 patients. Recent studies have shown that is an add-on tool, which has beneficial impact in the initial management of dyspneic patients especially to those with co-existed respiratory and heart disease. In suspected or confirmed COVID-19 cases, findings of interstitial sonographic pattern such as thick, irregular pleural line accompanied by focal B-lines and consolidation lesions are highly indicative of respiratory failure of pulmonary, non-cardiologic origin [28].

### 2.2. Undifferentiated Shock

One of the main challenges for the acute care physician is the management of the patient with undifferentiated shock. Prompt and accurate identification of the underlying cause is the cornerstone in order to reduce mortality.

Since there is no “gold standard” imaging modality, focused ultrasound protocols are designed to provide a global and structured approach regarding the classification of the shock, the identification of the primary etiology, and to guide a targeted resuscitation in order to optimize the outcome of the patient.

The most widely implementable protocols which include lung ultrasonography as a core view are the FALLS protocol (Fluid Administration Limited by Lung Sonography) created by Lichtenstein a few years ago [29], the widely accepted RUSH protocol (Rapid Ultrasound for Shock and Hypotension) [30], and the SHoC-hypotension protocol proposed by the International Federation for Emergency Medicine [31]. These protocols include lung scanning in order to assess the volume status of the patient by identifying pulmonary congestion if bilateral B lines and pleural effusions are detected. The combination of a low cardiac output and pulmonary congestion direct to the diagnosis of cardiogenic shock [29]. Additionally, a lung ultrasound can be used in conjunction with echocardiography for the estimation of fluid responsiveness in the shocked patient and could be a valuable hemodynamic monitoring tool and a guide to treatment by evaluating the filling status during fluid resuscitation [32].

All protocols include lung views in order to rule out alternative shock etiologies such as tension pneumothorax (obstructive shock), hypovolemia in the presence of a “dry” lung pattern (hypovolemic or distributive shock), and consolidation-infarct (obstructive shock) [33]. In the hemodynamically unstable patient, findings of dry, non-congested lungs and right ventricle strain in a combined bedside lung and echo scanning is highly suggestive of pulmonary embolism (PE). In addition, identification of a pleural-based hypoechoic parenchymal lesion is even more supportive for a PE related shock in the high risk patient. Thus, while resuscitation is ongoing, if pulmonary embolism is suspected, lung sonography can serve as an adjunct tool, without any delay or replacement of the gold standard diagnostic modality which is CT pulmonary angiogram.

**Figure 2:**
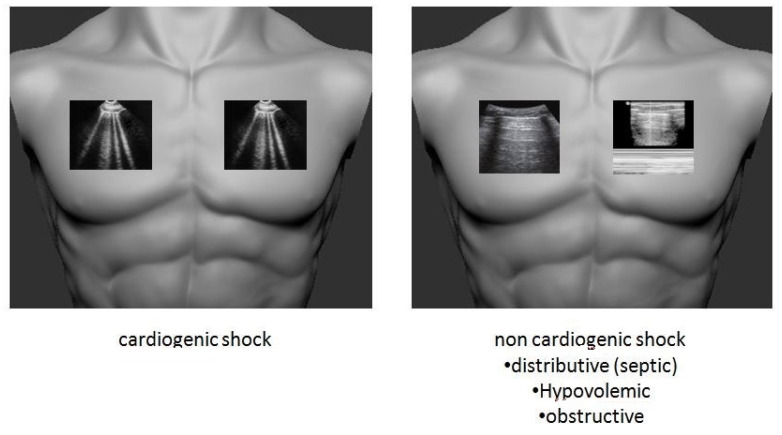
Lung sonography protocol for undifferentiated shock.

Proposed lung sonography-guided protocol for undifferentiated shock is shown in Figure 2.

### 2.3. Chest Pain

Chest pain is one of the most common clinical presentations in the acute cardiac care setting. Differential diagnosis is wide and includes life-threatening conditions of cardiac and non-cardiac origin. Lung sonography in addition to echo can help narrowing diagnostic dilemmas and set the diagnosis rapidly.

In the setting of acute coronary syndrome (ACS), lung scanning has the ability to identify clinically silent pulmonary edema and can serve as a prognostic and risk stratification tool. A study of Bedetti et al. has shown that the presence of B-lines is an independent predictor of adverse cardiac events and in-hospital mortality. Echocardiography findings of wall motion abnormalities or mechanical complications accompanied with pulmonary congestion on lung sonography are related with poor outcome [34,35].

In order to rule out lung pathologies, compared to traditional imaging modalities such as chest x-ray, lung scanning can readily and accurately diagnose pneumothorax by checking for lung sliding, pleural effusion, consolidation which could be an infarct, or pneumonia in the appropriate clinical setting [10,11].

**Figure 3:**
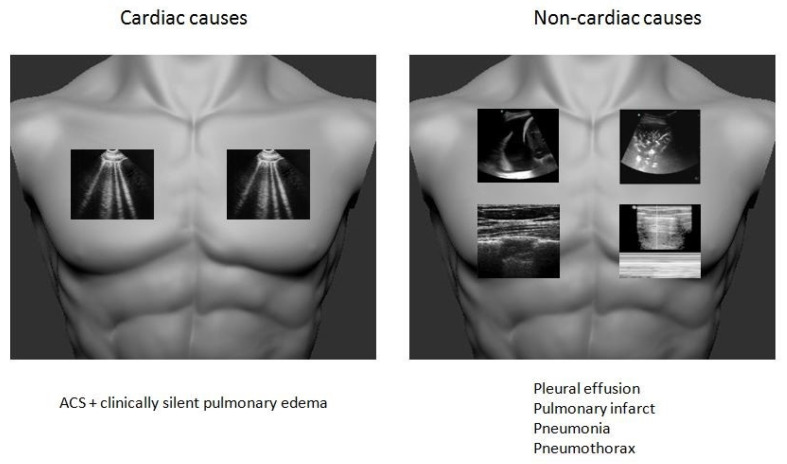
Lung sonographic decision making algorithm in chest pain. ACS: acute coronary syndrome.

A rapid decision making algorithm is shown in Figure 3.

### 2.4. Cardiac Arrest

One of the most common cardiovascular emergencies is cardiac arrest. An optimal outcome requires a structured and comprehensive management in a logical and timely manner. The use of ultrasound during cardiac arrest is well established (Advanced Life Support guidelines 2015) and lung ultrasonography is an essential component of all cardiac arrest ultrasound protocols in order to identify reversible causes mainly in pulseless electrical activity status [36,37]. Excluding tension pneumothorax is the first step, by assessing for lung sliding presence. Lung sonography is the recommended imaging modality, as it has a sensitivity of 92.3%, a specificity of 99.6%, a positive predictive value of 92.3%, and an accuracy of 99.3% in detecting pneumothorax [38]. Unilateral pleural effusion in the setting of trauma is highly suspicious for hemothorax and hypovolemia. A dilated left ventricle in echocardiography and concurrent detection of bilateral B-lines are indicative of cardiogenic shock in the appropriate clinical setting. In a high risk patient, identification of a triangular shape consolidation in combination with echocardiographic findings of right ventricular strain are indicative of massive PE as the cause of cardiac arrest [31,39].

**Figure 4:**
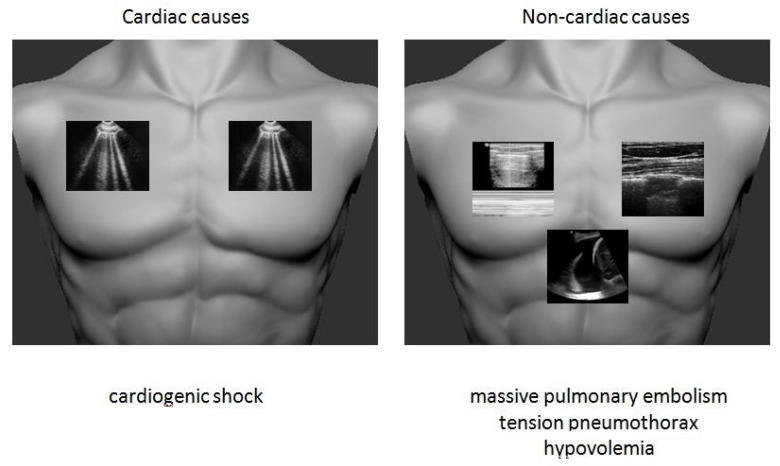
Lung sonographic approach in cardiac arrest.

A simplified and structured lung sonographic approach is shown in Figure 4.

## 3. Conclusions

Lung ultrasound is a non-invasive modality which can be repeated endlessly in real time, in order to narrow differential diagnosis in common undifferentiated clinical presentations in the acute cardiac care setting. Due to its dynamic power and evidenced superiority compared to conventional exams such as chest x-ray, it can be used as an adjunct to physical examination, echocardiography and biomarkers during the initial evaluation of patients presenting with time-sensitive diagnoses. Especially in those patients with comorbidities, lung scanning can easily rule in or rule out pulmonary pathologies which can mimic the clinical picture of cardiac emergencies. Thus, the modern cardiologist should consider lung sonography as a supportive tool for the holistic approach of the patient in the acute care setting.

## Data Availability

Data available in a publicly accessible https://pubmed.ncbi.nlm.nih.gov/.
